# Effects of Aspirin Eugenol Ester on Liver Oxidative Damage and Energy Metabolism in Immune-Stressed Broilers

**DOI:** 10.3390/antiox13030341

**Published:** 2024-03-13

**Authors:** Jiale Zhong, Wenrui Zhen, Dongying Bai, Xiaodi Hu, Haojie Zhang, Ruilin Zhang, Koichi Ito, Yi Zhang, Bingkun Zhang, Yanbo Ma

**Affiliations:** 1 Department of Animal Physiology, College of Animal Science and Technology, Henan University of Science and Technology, Luoyang 471000, China; zhongjiale@stu.haust.edu.cn (J.Z.); 9900427@haust.edu.cn (D.B.); ms.xiaodihu@stu.haust.edu.cn (X.H.); zhanghaojie@stu.haust.edu.cn (H.Z.); ruilinzhang@stu.haust.edu.cn (R.Z.); 9906319@haust.edu.cn (Y.Z.); 2 Henan International Joint Laboratory of Animal Welfare and Health Breeding, College of Animal Science and Technology, Henan University of Science and Technology, Luoyang 471000, China; 3 Department of Food and Physiological Models, Graduate School of Agricultural and Life Sciences, The University of Tokyo, Ibaraki 305-8577, Japan; akoito@mail.ecc.u-tokyo.ac.jp; 4 State Key Laboratory of Animal Nutrition, Department of Animal Nutrition and Feed Science, College of Animal Science and Technology, China Agricultural University, Beijing 100000, China; zhangbk@cau.edu.cn; 5 Innovative Research Team of Livestock Intelligent Breeding and Equipment, Longmen Laboratory, Luoyang 471000, China

**Keywords:** aspirin eugenol ester, immune stress, broilers, oxidative damage, energy metabolism

## Abstract

The aim of this study was to investigate the effects of aspirin eugenol ester (AEE) on liver oxidative damage and energy metabolism in immune-stressed broilers. In total, 312 broilers were divided into 4 groups (saline, LPS, SAEE, and LAEE). Broilers in the saline and LPS groups were fed a basal diet; the SAEE and LAEE groups had an added 0.01% AEE in their diet. Broilers in the LPS and LAEE groups were injected with lipopolysaccharides, while the saline and SAEE groups were injected with saline. Results showed that AEE increased the body weight, average daily gain, and average daily feed intake, as well as decreasing the feed conversion ratio of immune-stressed broilers. AEE protects against oxidative damage in immune-stressed broiler livers by elevating the total antioxidant capacity, superoxide dismutase activity, and glutathione S-transferase alpha 3 (*GSTA3*) and glutaredoxin 2 (*GLRX2*) expression, while decreasing malondialdehyde content. AEE lessened inflammation by reducing prostaglandin-F2α production and prostaglandin-endoperoxide synthase 2 (*PTGS2*) and interleukin-1beta (*IL-1β*) expression. AEE decreased oxidative phosphorylation rates by increasing succinic acid levels and lowering both adenosine diphosphate (ADP) levels and ceroid lipofuscinosis neuronal 5 (*CLN5*) expression. AEE modulated the metabolism of phenylalanine, tyrosine, lipids, and cholesterol by reducing the phenyllactate and L-arogenate levels, lowering dopachrome tautomerase (*DCT*) and apolipoprotein A4 (*APOA4*) expression, and increasing phenylpyruvic acid and dopa decarboxylase (*DDC*) expression. In summary, AEE can effectively alleviate liver oxidative damage and energy metabolism disorders in immune-stressed broilers.

## 1. Introduction

Intensive farming seems to have become the norm for guaranteeing the efficient production of livestock and poultry. However, while intensive farming results in large-scale economies, it is subject to some serious problems, such as high feeding density, the need for frequent vaccination, improper management, a stressful environment, and a rapid increase in pathogenic infections, all of which lead to immune stress in livestock and poultry, through the loss of homeostasis [1,2,3]. Immune stress not only reduces the growth performance and antioxidant capacity of broilers, but also generates ROS through the production of excessive pro-inflammatory cytokines, resulting in oxidative damage [4,5,6,7]. In addition, immune stress can cause an imbalance in energy production, by changing the metabolism of amino acids and glycerol phospholipids in the livers of broilers [8]. Despite these studies, the mechanism of the effects of immune stress on oxidative damage and energy metabolism in broilers has not been fully elucidated; the question remains of how to effectively alleviate this immune stress-related oxidative damage and energy imbalance in broilers.

Aspirin eugenol ester (AEE) is a chimeric, non-steroidal anti-inflammatory drug made from aspirin and eugenol. It has a more stable structure than either compound alone, causes less irritation to the gastrointestinal tract, and possesses a wide range of pharmacological activity as an as anti-inflammatory, antioxidant, antipyretic, and analgesic [9]. After comprehensive evaluation of the pharmacodynamics, pharmacokinetics, and toxicology, it was confirmed that AEE was safe for widespread use in poultry production [10,11,12,13,14]. Several studies verified AEE’s function as an antioxidant and adjunct in energy metabolism. AEE mitigates the liver damage caused by paraquat through a reduction in oxidative stress, the restoration of regular metabolic processes, and the preservation of mitochondrial activity [15,16]. AEE ultimately alleviated lipopolysaccharides (LPS)-induced acute lung injury in rats by reducing inflammatory responses, decreasing oxidative damage, and regulating energy metabolism [17]. The potential mechanism of AEE’s influence on hyperlipidemia in rats was related to the regulation of the metabolism of amino acids, energy, glycerophospholipids, and glutathione [18]. However, until now, the effects of feeding AEE to immune-stressed broilers have rarely been investigated. Therefore, this research aimed to explore the effects of AEE on liver oxidative damage and energy metabolism in immune-stressed broilers by assessing the levels of enzymes related to the antioxidant, metabolite, and transcriptome profiles of the liver. The results provide a theoretical basis for the use of AEE in improving poultry agriculture.

## 2. Materials and Methods

### 2.1. Diets and Broiler Chickens

LPS was purchased from Sigma-Aldrich Trading Co. (St. Louis, MO, USA). The dosage and method of administering LPS were derived from earlier research [19]. AEE was obtained from the Lanzhou Veterinary Research Institute. For the experimental diet, AEE was present during the entire period and a preliminary experiment was performed to determine the optimal concentration of AEE in the feed, 0.01%. The basic diet was purchased from Fanda Feed Co. (Luoyang, China). and the composition and nutritional level of the basic diet were the same as in a previous study [5]. Male Aiba Yijia (Arbor Acres) broilers were purchased from Quanda Poultry Breeding Co. (Henan, China).

### 2.2. Experimental Procedures

A total of 312 AA male broilers were randomly divided into 4 groups, with 6 replicates for each group and 13 broilers for each replicate. The broilers in both the saline and LPS groups received a standard diet and broilers in the SAEE and LAEE groups were fed diets supplemented with 0.01% AEE. Birds in the LPS and LAEE groups were intraperitoneally injected with LPS, while those in both the saline and SAEE groups received an intraperitoneal injection of an identical saline volume for 3 consecutive days, starting at the age of 14 days.

### 2.3. Production Performance

Measurements of body mass and feed consumption in each cage of broilers were taken at the ages of 14, 15, and 17 days. The body weight (BW) was taken on day 17, as well as the average daily gain (ADG), average daily feed intake (ADFI), and the feed conversion ratio (FCR) on days 14 through 17 being calculated.

### 2.4. Sample Collection

Sampling was conducted four times, scheduled at 2, 4, 24, and 72 h post the intraperitoneal injection on the 14th day. One broiler was selected from every duplicate each time; that is, 6 broilers were selected from each group, a total of 24 broilers. First of all, the blood was collected from the wing vein of the broilers. The collection of four-gram liver specimens from each broiler’s hepatic lobule center after the blood collection was completed. The gathered blood underwent centrifugation at 3500 rpm for fifteen minutes, followed by the collection and preservation of the serum at −80 °C. The gathered liver specimens were rapidly frozen using liquid nitrogen and were preserved at a temperature of −80 °C.

### 2.5. Determination of Oxidative Damage Indices

The measurement of total protein (TP), total antioxidant capacity (T-AOC), superoxide dismutase (SOD) activity, and malondialdehyde (MDA) concentration were conducted using various assays including a total protein quantitative assay (A045-2-2), a total antioxidant capacity assay (A015-2-1), a superoxide dismutase assay (A001-3-2), and a malondialdehyde assay (A003-1-2). All kits originate from Nanjing Jiancheng Biotechnology Co., Ltd. (Nanjing, China), with all procedures strictly adhering to the provided instructions.

### 2.6. Metabolome Assay and Data Analysis 

**Figure 1:**
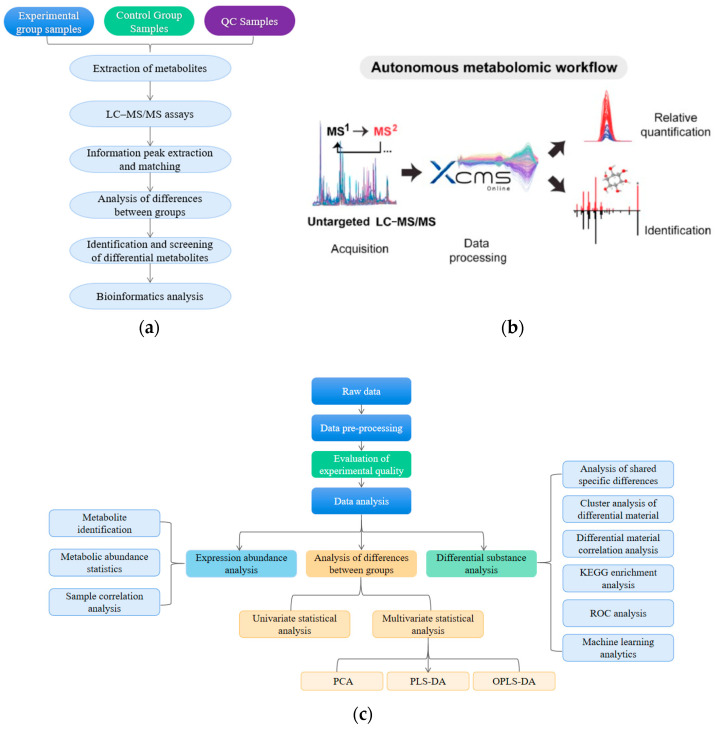
The metabolomics assay process. (**a**) Main steps of the experimental process; (**b**) Metabolite structure identification, data preprocessing, and experimental data quality evaluation; (**c**) Main steps of the data analysis.

The metabolomics assay process is shown in Figure 1a and mainly includes metabolite extraction, quality control (QC) sample preparation, procedures for liquid chromatograph-mass spectrometer (LC–MS/MS), and the analysis of mass spectrometry data. The method of sample preparation refers to a previous study [20]. QC samples were prepared by taking a portion of the extracted sample to be tested and mixing it with a QC sample. The chromatographic conditions for on-line detection using a Thermo Vanquish (Thermo Fisher Scientific, Waltham, MA, USA) ultra-high performance liquid chromatography (UPLC) system, including an Acquity UPLC^®^ HSS T3 column (2.1 × 100 mm, 1.8 µm) (Waters, Milford, MA, USA) with a flow rate of 0.3 mL/min, a temperature of 40 °C, and an injection volume of 2 μL [21]. To conduct mass spectrometry, a mass spectrometer of the Thermo Orbitrap Exploris 120, equipped with an electrospray ionization source (ESI), was utilized. Data gathering occurred in both positive and negative ion modes [22]. ProteoWizard transformed the raw data into mzXML format, utilizing XCMS software (https://xcmsonline.scripps.edu/) for aligning peaks, adjusting retention times, and isolating peak regions. Initially, the information derived from XCMS extraction underwent an identification of the metabolite structures and subsequent data preprocessing (Figure 1b), followed by experimental data quality evaluation, and lastly data analysis (Figure 1c), which included both univariate and multidimensional statistical analysis, differential metabolite screening and correlation analysis, machine learning, ROC analysis, and KEGG pathway analysis.

### 2.7. Transcriptome Determination and Data Analysis

Using oligo (dT) magnetic beads, polyA mRNA was extracted from the total RNA and subsequently segmented into approximately 300 bp pieces through ionic interruption. For the initial strand of cDNA synthesis, a 300 bp fragment was chosen, serving as a blueprint for creating the subsequent strand of cDNA. Once the library was finalized, the fragments underwent PCR amplification and were chosen according to their size, approximately 450 base pairs. Subsequently, an Agilent 2100 bioanalyzer was employed to verify the quality of the libraries, and those with varying index sequences were combined in proportion to their effective concentration and the necessary data volume. The combined libraries were evenly diluted to a concentration of 2 nM and were then alkali denatured to create single-stranded libraries. The libraries underwent PE sequencing utilizing both NGS and Illumina sequencing systems.

Filtering was applied to the unprocessed data, followed by a comparison of the refined filtered data with the species’ reference genome. The expression levels of each gene were determined using the outcomes of the comparison. Subsequently, the samples underwent additional analysis for differential expression, enrichment, and cluster analysis. Lastly, the corresponding reads were spliced to restore the transcript sequence.

### 2.8. Conjoint Analysis of Transcriptomics and Metabolomics Data 

First, correlation and O2PLS analysis were performed on the quantitative assay results of metabolomics and transcriptomics; then, differentially expressed metabolites and transcript information were extracted. Transcripts linked to pertinent enzymes were isolated by examining the correlation analysis outcomes derived from the metabolite data in the KEGG database. The data were organized according to changing trends of the differential metabolites and transcripts with corresponding relationships, significant enrichment pathways common to metabolomics and transcriptomics, and pathways for the co-enrichment of differential metabolites and differentially expressed genes.

### 2.9. Verification of Quantitative Real-Time PCR Results 

Using TRIzol reagent (Invitrogen company, Carlsbad, CA, USA), total RNA was extracted from broiler livers and its quality was evaluated by measuring its absorbance at 260 nm and 280 nm and calculating the 260/280 ratio. The creation of cDNA was carried cDNA synthesis kit (Toyobo, Osaka, Japan). Table 1 displays the primers for both the target genes and the gene for *HPRT* loading control. The quantitative real-time PCR process utilized a Toyobo kit for real-time PCR within a Roche LightCycle device (Shanghai, China). The analysis of the data was conducted using the 2^−∆∆CT^ technique.

### 2.10. Statistical Analysis

All statistical evaluations were conducted using SPSS 20.0 and the graphs were created using Graphpad Prism 8.0. The mean values of each group were compared using a one-way ANOVA. Findings are presented as an average plus or minus the standard error. A *p* value less than 0.05 is indicative of the data’s statistical significance.

## 3. Results

### 3.1. Production Performance

The production performance results are shown in Table 2. In contrast to the saline group, there was a notable reduction in BW (day 17), ADG, and ADFI, while the broilers’ FCR presented a substantial increase during LPS-induced immune stress (*p* < 0.05). Solely adding AEE showed no notable impact on production performance (*p* > 0.05). In contrast to the LPS group, there was a substantial increase in BW, ADG, and ADFI, while the FCR showed a notable reduction in the broilers in the LAEE group (*p* < 0.05). 

### 3.2. Antioxidant Function

The measurements of antioxidant capacity in broiler livers are shown in Table 3. In contrast to the saline group, a significant decrease in T-AOC levels and SOD activity was observed in the broilers of the LPS group on days 14, 15, and 17, while the MDA levels increased (*p* < 0.05). Solely adding AEE showed no notable impact on the antioxidant capacity of broiler livers (*p* > 0.05). In contrast to the LPS group, a significant increase in T-AOC levels and SOD activity was observed in the broilers of the LAEE group on days 14, 15, and 17, while the MDA levels decreased. (*p* < 0.05).

The measurements of antioxidant capacity in broiler serum are shown in Table 4. In contrast to the saline group, the T-AOC level decreased significantly in the broiler serum of the LPS group on days 15 and 17, the SOD activity decreased significantly on days 14, 15, and 17, and the content of MDA increased significantly on day 17 (*p* < 0.05). Solely adding AEE showed no notable impact on the antioxidant capacity of broiler serum (*p* > 0.05). In contrast to the LPS group, the T-AOC level increased significantly in the broiler serum of the LAEE group on days 15 and 17, the SOD activity increased significantly on days 14, 15, and 17, and the content of MDA decreased significantly on day 17 (*p* < 0.05).

### 3.3. Metabolomic Analysis

**Figure 2:**
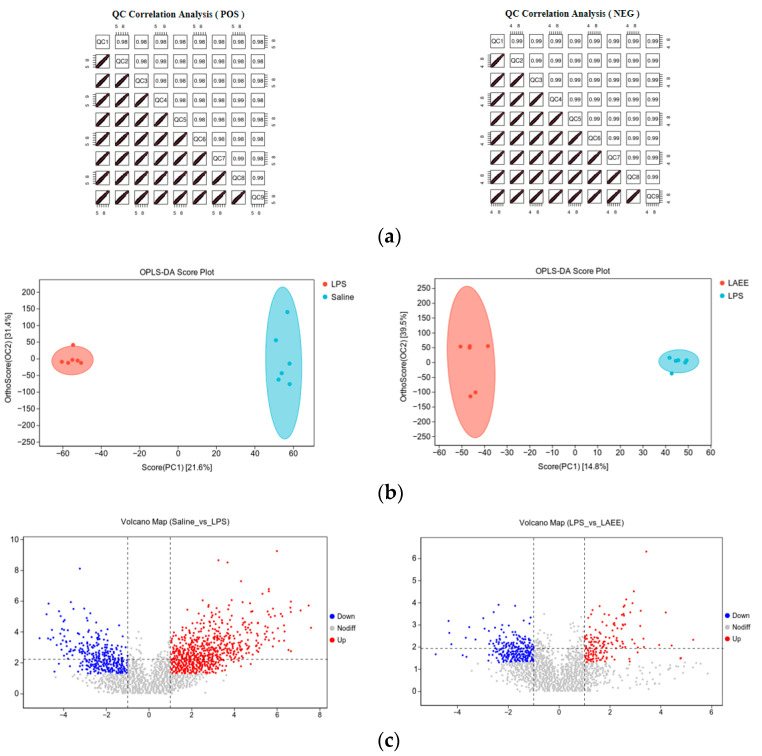
The metabolic state of liver samples of broilers in the different treatment groups. (**a**) QC correlation analysis of broiler livers. The median value of each cell is the correlation coefficient; a coefficient > 0.9 is considered to be extremely well correlated. (**b**) Analysis of LC–MS/MS using OPLS-DA. Principal component 1 is PC1, PC2 is principal component 2, and differently colored points and ellipses represent samples and confidence intervals for different groupings. (**c**) Graph of a volcano. Metabolites showing notable differences; those with FC > 1 and *p* values below 0.05 are marked in red, while those with FC < 1 and *p* values under 0.05 are shown in blue. Differential metabolites of non-significance are marked in gray.

**Figure 3:**
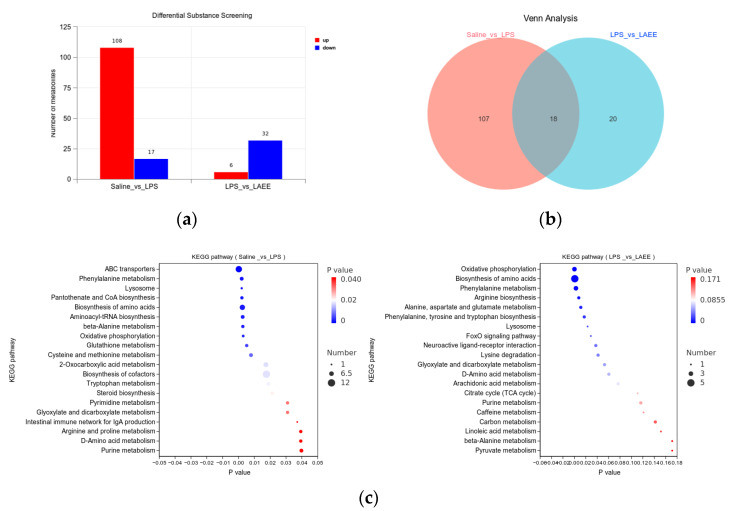
Comparative analysis of differential metabolites and differential metabolic pathways in the livers of broilers from different treatment groups. (**a**) Histogram of differential metabolites. (**b**) Venn diagram of differential metabolites. (**c**) Factor diagram of KEGG enrichment analysis. The enrichment degree of each pathway was analyzed by *p* value and the number of metabolites. The dimensions of the bubble indicate the count of metabolites. The varying hues, ranging from red to blue, symbolize the magnitude of the *p* value. A lower *p* value enhances the significance of the enrichment level.

The results of the QC sample test are shown in Figure 2a. The liver samples from each group were well correlated and a clear separation was observed in the OPLS-DA score plot (Figure 2b). The changes in metabolite profiles are shown in Figure 2c. Significant differences in up-regulated metabolites are shown in red, while blue represents significant differences in down-regulated metabolites (*FC* < 1, *p* < 0.05). The number of specific differential metabolites that are significantly produced is shown in Figure 3a. In contrast to the saline group, the LPS group’s livers exhibited 125 metabolites produced differently, with 108 showing increased expression and 17 showing decreased expression. In contrast to the LPS group, 38 distinct metabolites were detected in the LAEE group, with 6 experiencing an increase and 32 experiencing a decrease. There were 18 differential metabolites in common between the two comparison groups (Figure 3b).

To comprehensively evaluate the metabolic pathway in broiler livers, the screened differential metabolites were subjected to KEGG enrichment analysis (Figure 3c). In contrast to the saline group, the differential metabolic pathways in livers of the LPS group mainly included twelve kinds of amino acid metabolism (phenylalanine, β-alanine, glutathione, cysteine, methionine, tryptophan, arginine, proline, D-amino acid, valine, leucine, and isoleucine), oxidative phosphorylation, glyoxylic acid, dicarboxylic acid and 2-oxocarboxylic acid metabolism, pyrimidine and purine metabolism, lysosome formation, and steroid biosynthesis (*p* < 0.05). In contrast to the LPS group, the differential metabolic pathways in the livers of the LAEE group mainly included oxidative phosphorylation, phenylalanine metabolism, and lysosome formation (*p* < 0.05). The types of differential metabolites in the differential metabolic pathways are shown in Table 5 and Table 6. In contrast to the saline group, the contents of adenosine diphosphate (ADP), L-tyrosine, trans-cinnamate, phenyllactate, and prostaglandin-F2α in the livers of the LPS group were significantly increased, while the concentration of succinic acid decreased significantly (*p* < 0.05). In contrast to the LPS group, the contents of ADP, phenyllactate, L-arogenate, and prostaglandin-F2α in the livers of the LAEE group decreased significantly, while the concentration of phenylpyruvic acid and succinic acid increased significantly (*p* < 0.05).

### 3.4. Conjoint Analysis of Transcriptomics and Metabolomics Data

**Figure 4:**
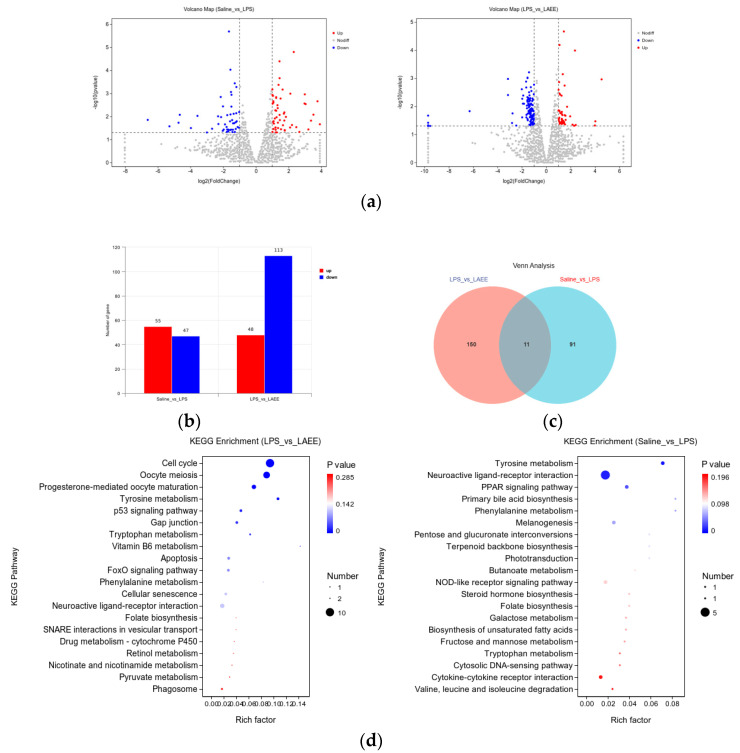
Comparative analysis of differential genes and differential pathways in the livers of broilers from different treatment groups. (**a**) Graph of a volcano. (**b**) Histogram of differential genes. (**c**) Venn diagram of differential genes. (**d**) Factor diagram of KEGG enrichment analysis. The enrichment degree of each pathway was analyzed by *p* value and the number of genes. The dimensions of the bubble indicate the count of genes. The varying hues, ranging from red to blue, symbolize the magnitude of the *p* value. A lower *p* value enhances the significance of the enrichment level.

The changes of genes are shown in Figure 4a, with red representing up-regulated differentially expressed genes (DEGs) and blue indicating down-regulated DEGs (*FC* < 1, *p* < 0.05). The specific number of DEGs is shown in Figure 4b. In contrast to the saline group, out of the 102 DEGs examined in the LPS group’s livers, 55 showed an increased expression and 47 showed a decreased expression. Differing from the LPS group, 161 differentially expressed genes (DEGs) were examined in the LAEE group’s livers, with 48 showing an increased expression and 113 showing a decreased expression. There were 18 common DEGs between the two comparison groups (Figure 4c). In order to comprehensively evaluate the differences in pathways, the selected DEGs were analyzed using the KEGG enrichment database and the first 20 pathways were obtained (Figure 4d).

**Figure 5:**
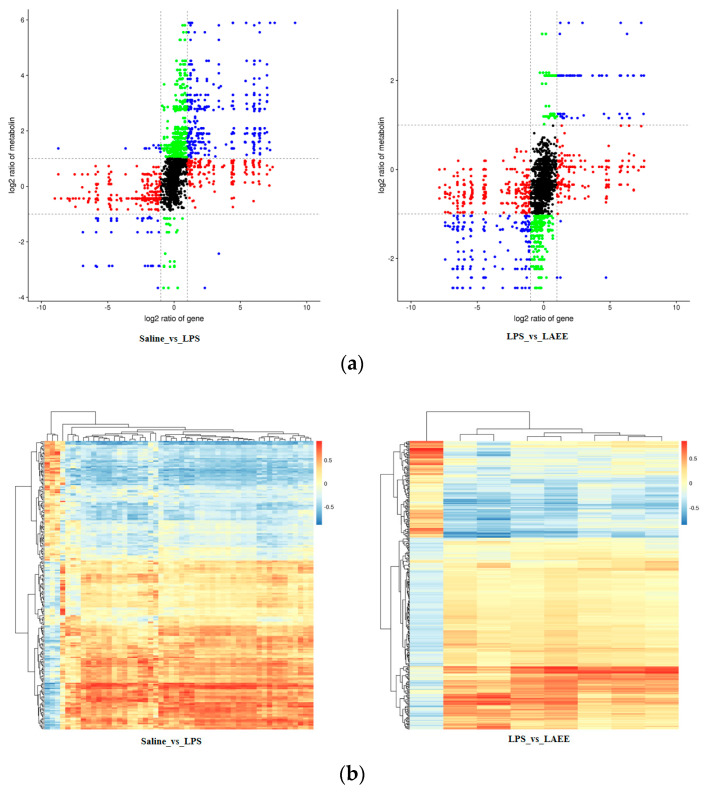
Conjoint analysis of transcriptomics and metabolomics data. (**a**) Nine-quadrant plot. Abscissa is the diversity of genetic differences, ordinate is the diversity of metabolites, and different colors indicate different trends. (**b**) Cluster heat map of differential genes and differential metabolites; metabolites are displayed vertically, and the color represents the correlation coefficient.

The correlation between differential genes and metabolites is shown in Figure 5a,b, where a strong correlation between metabolites and transcripts was found. The differential metabolites and transcripts were mapped to the KEGG pathway database at the same time and the differential genes related to differential metabolites and differential metabolic pathways were organized according to the mapped pathway information. The screening results of the related DEGs showed that, in contrast to the saline group, the mRNA expression of *PTGS2*, *IL-1β*, *CLN5*, *DCT*, and *APOA4* in the livers of the LPS group increased significantly, while the mRNA expression of *DDC*, *GSTA3*, and *GLRX2* decreased significantly (*p* < 0.05). In contrast to the LPS group, the mRNA expression of *PTGS2*, *IL-1β*, *CLN5*, *DCT*, and *APOA4* in the livers of the LAEE group decreased significantly, while the mRNA expression of *DDC*, *GSTA3*, and *GLRX2* increased significantly (*p* < 0.05).

### 3.5. Quantitative Real-Time-PCR Validation of DEGs

**Figure 6:**
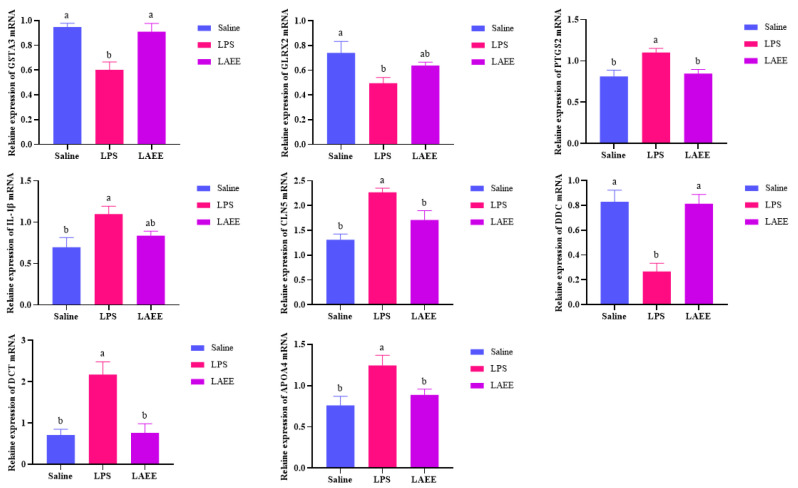
qRT-PCR validation of DEGs. Different letters indicate statistical significance (*p* < 0.05). The analysis of the data was conducted using a one-way ANOVA. Each value represents the average plus or minus the standard error (SE), with six broilers in each group.

The qRT-PCR results are shown in Figure 6. The expression of eight genes is consistent with the trends described above for the transcriptomic analysis of the livers, thus further confirming the reliability of the transcriptomics data.

## 4. Discussion

Although many studies have reported the effects of immune stress on oxidative damage and energy metabolism [6,7,23,24], how to effectively alleviate this immune stress-related oxidative damage and energy imbalance in broilers remains to be investigated. AEE exhibits multiple pharmacological properties, including anti-inflammatory, antioxidant, and metabolic control, and has been verified as safe for poultry farming [9,11,12]. AEE proposes significant potential for use in alleviating immune stress among broilers. Therefore, the purpose of this study is to explore the effects of AEE on liver oxidative damage and energy metabolism in immune-stressed broilers.

Immune stress affects the growth of broilers and reduces both feed intake and body weight gain [25,26]. The results of this study showed that immune stress resulted in a significant decrease in BW, ADG, and ADFI and a significant increase in FCR, which is consistent with the results of previous studies [27,28]. AEE significantly increased BW, ADG, and ADFI and significantly reduced FCR in immune-stressed broilers; thus, the dietary addition of AEE could effectively alleviate the decrease in the growth performance caused by immune stress.

Immune stress decreases antioxidant activity, and the inflammatory response caused by immune stress increases ROS production, which causes an imbalance between the antioxidant system and ROS production, leading to oxidative stress and damage [23,29,30,31,32]. A variety of metrics are employed to assess antioxidant abilities and oxidative damage. The T-AOC reflects the ability of cells to scavenge ROS, primarily by SOD, the key enzyme in the antioxidant defense system. MDA is an end-product of lipid peroxidation and can be used as a biomarker for evaluating the degree of lipid peroxidation. MDA content is positively correlated with ROS activity [33,34,35]. In this study, the T-AOC and SOD activity in the livers and serum of immune-stressed broilers were decreased, the mRNA expression of the antioxidant-related genes, *GSTA3* and *GLRX2*, was down-regulated, and the MDA content was increased. The decreased expression of the glutathione transferase gene, *GSTA3*, caused by immune stress may increase ROS [36]. *GLRX2* is the gene encoding the mitochondrial antioxidant, Grx2, and its absence prevents the compensatory recovery of the redox system and increases cellular oxidation [37]. AEE possesses a variety of pharmacological activities including anti-inflammatory and antioxidant, which can effectively reduce the inflammatory response and oxidative damage [38,39]. Our results showed that AEE enhanced the T-AOC and SOD activity, increased *GSTA3* and *GLRX2* mRNA expression, and reduced MDA concentration, which is consistent with previous studies [17]. Furthermore, AEE successfully counteracted the notable rise in prostaglandin-F2α and mRNA levels of *PTGS2* and *IL-1β* in broiler livers, which were induced by immune stress. This aligns with findings from earlier studies [17,40,41]. Prostaglandin-F2α is synthesized from arachidonic acid by *PTGS2* (COX-2) and is capable of triggering an inflammatory response [42,43,44]. The overexpression of IL-1 β can also lead to inflammation [45]. In summary, AEE reduced oxidative damage in immune-stressed broilers by improving the antioxidant capacity and reducing inflammatory responses and ROS production.

Immune stress can lead to inflammation, oxidative stress, and an increased metabolic rate, all of which require energy to preserve bodily equilibrium [8,46,47,48]. This research revealed a notable shift in the oxidative phosphorylation process in the broiler livers of the LPS group, in contrast to the saline group, marked by a substantial reduction in succinic acid levels and a notable rise in ADP content and *CLN5* mRNA expression. A reduction in succinic acid levels, coupled with a rise in ADP content and *CLN5* mRNA expression signals an escalation in the rate of oxidative phosphorylation and ATP generation [49,50,51,52]. Adding dietary AEE markedly counteracted the impact of immune stress on broilers’ liver oxidative phosphorylation pathways. In conclusion, immune stress accelerates oxidative phosphorylation in broiler livers, enhancing ATP production to sustain bodily equilibrium, and AEE can significantly reduce energy consumption in immune-stressed broilers. Interestingly, ROS are byproducts of oxidative phosphorylation in the respiratory chain [53,54,55] and an increase in the rate of oxidative phosphorylation leads to an increase in ROS production [56,57,58]. Therefore, the protective effect of AEE on liver oxidative damage in immune-stressed broilers may be related to its decrease in the rate of oxidative phosphorylation.

Immune stress changes the metabolism in animals, enabling re-distribution of nutrients to support immune functions [59,60]. In the present study, significant changes in the metabolic pathways associated with phenylalanine were observed in the livers of immune-stressed broilers, in which the metabolites, L-tyrosine, trans-cinnamate, and phenyllactate were significantly increased, which is consistent with previous studies [24,61]. L-tyrosine, trans-cinnamate, and phenyllactate are the catabolic products of phenylalanine and their increase represents the enhancement of phenylalanine catabolism [62,63,64]. AEE treatment caused a significant increase in the amount of phenylpyruvic acid and significant decreases in the amount of phenyllactate and L-arogenate. Phenylpyruvic acid is an intermediate in phenylalanine metabolism, which can be transformed with phenylalanine and phenyllactate, the increase in phenylpyruvic acid content and decrease in phenyllactate content indicate the decrease in the catabolism of phenylalanine [65,66,67]. L-arogenate can be converted to aromatic amino acids such as phenylalanine and phenylpyruvic acid [68]. Therefore, AEE effectively inhibits the increased phenylalanine catabolism in the livers of immune-stressed broilers. In addition, AEE effectively blocks the decrease in *DDC* expression and the increase in *DCT* expression. *DDC* is a key gene in melanin production from L-tyrosine, and a *DDC* deletion will interrupt melanin synthesis and lead to L-tyrosine accumulation [69]. *DCT* is the rate-limiting gene for melanin production, and it is activated when melanin synthesis is blocked [70,71]. As a result, in this study, the increase in L-tyrosine in immune-stressed broilers’ livers was caused by an increase in phenylalanine catabolism and a decrease in L-tyrosine catabolism. Interestingly, the accumulation of L-tyrosine leads to a compensatory increase in succinate dehydrogenase activity in the liver [63], which is consistent with the increase in L-tyrosine and the decrease in succinic acid seen in this study, so L-tyrosine metabolism is closely related to the oxidative phosphorylation pathway. Moreover, AEE increased the expression of *APOA4* in the liver. *APOA4* can promote cholesterol metabolism, and the overexpression of *APOA4* can lead to the inhibition of lipogenesis, increased fat decomposition, and the oxidation of fatty acids [72,73]. In summary, AEE can effectively relieve the enhancement of phenylalanine catabolism, the decrease in tyrosine catabolism, and the disordered lipid and cholesterol metabolism in immune-stressed broilers.

## 5. Conclusions

In the present study, we report for the first time that AEE can improve growth performance in immune-stressed broilers by altering the transcriptome and metabolite profiles in the liver. AEE reduces oxidative damage in immune-stressed broilers by improving the antioxidant capacity and reducing the oxidative phosphorylation rate, inflammatory responses, and ROS production. AEE also improves energy metabolism in immune-stressed broilers through altering phenylalanine and tyrosine metabolism, as well as lipid and cholesterol metabolism. Our results provide new insights into the use of AEE to relieve immune stress in broilers through improving liver oxidative damage and energy metabolism.

## Figures and Tables

**Table 1:** The primer sequences used for real-time PCR.

Target Gene ^1^	Forward Primer, Reverse Primer (5′-3′) ^2^	GenBank Number
*HPRT*	F: CAGCCCCTGCATCGTGATTG	NM_204848.2
	R: TTCACGTGCCAGTCTCTCTG	
*GSTA3*	F: CATCCTCAACTACATAGCA	NM_001001777.2
	R: CAGTCCTTCCACATACAT	
*GLRX2*	F: ACTGCGTGGTGATTTTCTCT	XM_004943130.5
	R: CCACCCGTCATCTGTTCAAG	
*PTGS2*	F: CCGAATCGCAGCTGAATTCA	XM_040704521.2
	R: GAAAGGCCATGTTCCAGCAT	
*IL-1β*	F: TGGGCATCAAGGGCTACAAG	XM_015297469.3
	R: AGGCGGTAGAAGATGAAGCG	
*CLN5*	F: GACTCTTACGAATGCTCTAA	XM_046910089.1
	R: TAGGTTCTCCGCTGAATA	
*DDC*	F: TTGACTGCTCTGCTATGTG	XM_419032.8
	R: AGACTCCTGGTGGTGATG	
*DCT*	F: AAGCAGAATGGAATGGAA	XM_025144675.3
	R: TCTCTATTGTATGTCTTCTTCA	
*APOA4*	F: GCAAGCTGAGATCACCAA	NM_204938.3
	R: TCCTCGGCGTATGAGTTC	

^1^*GSTA3*: glutathione S-transferase alpha 3; *GLRX2*: glutaredoxin 2; *PTGS2*: prostaglandin-endoperoxide synthase 2; *IL-1β*: interleukin-1beta; *CLN5*: ceroid lipofuscinosis neuronal 5; *DDC*: dopa decarboxylase; *DCT*: dopachrome tautomerase; *APOA4*: apolipoprotein A4. ^2^ F: forward primer; R: reverse primer.

**Table 2:** Comparative analysis of growth performance of broilers from different treatment groups.

Items ^1^	Saline	LPS	SAEE	LAEE	SEM	*p* Value
BW (g)	530.69 ^a^	491.42 ^b^	532.63 ^a^	516.76 ^a^	4.471	0.00
ADG (g)	47.24 ^a^	35.49 ^b^	46.74 ^a^	44.15 ^a^	0.944	0.00
ADFI (g)	62.07 ^a^	52.67 ^b^	61.65 ^a^	60.04 ^a^	0.678	0.00
FCR	1.31 ^b^	1.50 ^a^	1.32 ^b^	1.36 ^b^	0.021	0.02

^1^ BW: body weight; ADG: average daily gain; ADFI: average daily feed intake; FCR: feed conversion ratio. Numerical values with different letters differed significantly.

**Table 3:** Comparative analysis of antioxidant function of broiler livers in different treatment groups.

Items ^1^	Saline	LPS	SAEE	LAEE	SEM	*p* Value
T-AOC (mmol/g)						
14 d (2H)	0.18	0.16	0.18	0.18	0.005	0.18
14 d (4H)	0.18 ^a^	0.15 ^b^	0.18 ^a^	0.18 ^a^	0.003	0.01
15 d	0.19 ^a^	0.16 ^b^	0.19 ^a^	0.18 ^a^	0.004	0.04
17 d	0.21 ^a^	0.17 ^b^	0.21 ^a^	0.20 ^a^	0.005	0
SOD (U/mgprot)						
14 d (2H)	26.79	24.09	26.42	27.04	0.315	0.16
14 d (4H)	27.58 ^a^	24.36 ^b^	26.33 ^a^	26.53 ^a^	0.372	0.02
15 d	27.01 ^a^	23.60 ^b^	26.33 ^a^	26.07 ^a^	0.472	0.04
17 d	23.75 ^a^	20.09 ^b^	24.84 ^a^	23.41 ^a^	0.426	0.03
MDA (nmol/mgprot)						
14 d (2H)	0.49	0.54	0.49	0.46	0.016	0.46
14 d (4H)	0.44 ^b^	0.68 ^a^	0.47 ^b^	0.48 ^b^	0.031	0.03
15 d	0.39 ^b^	0.51 ^a^	0.37 ^b^	0.39 ^b^	0.02	0.04
17 d	0.37 ^b^	0.52 ^a^	0.39 ^b^	0.38 ^b^	0.021	0.02

^1^ T-AOC: total antioxidant capacity; SOD: superoxide dismutase; MDA: malondialdehyde. Numerical values with different letters differed significantly.

**Table 4:** Comparative analysis of antioxidant function of broiler serum in different treatment groups.

Items ^1^	Saline	LPS	SAEE	LAEE	SEM	*p* Value
T-AOC (mmol)						
14 d (2H)	0.94	0.89	0.93	0.85	0.018	0.22
14 d (4H)	1.12	0.97	1	1.11	0.03	0.18
15 d	1.21 ^a^	1.10 ^b^	1.21 ^a^	1.22 ^a^	0.015	0.01
17 d	1.24 ^a^	1.10 ^b^	1.20 ^a^	1.21 ^a^	0.016	0.01
SOD (U/mL)						
14 d (2H)	10.62	9.84	11.63	10.34	0.485	0.62
14 d (4H)	13.56 ^a^	10.51 ^b^	12.24 ^ab^	12.15 ^ab^	0.451	0.12
15 d	12.41 ^a^	9.56 ^b^	11.68 ^a^	11.95 ^a^	0.37	0.02
17 d	11.51 ^a^	9.28 ^b^	10.50 ^ab^	10.70 ^a^	0.311	0.02
MDA (nmol/mL)						
14 d (2H)	1.4	1.39	1.36	1.3	0.066	0.96
14 d (4H)	1.02	1.15	1.1	1	0.034	0.36
15 d	1.66	1.95	1.61	1.71	0.071	0.35
17 d	1.58 ^b^	2.19 ^a^	1.79 ^b^	1.77 ^b^	0.044	0.01

^1^ T-AOC: total antioxidant capacity; SOD: superoxide dismutase; MDA: malondialdehyde. Numerical values with different letters differed significantly.

**Table 5:** Examining the varied metabolites and associated pathways in the livers between the saline and LPS groups.

Metabolite	*p* Value	Variation	Pathway
Succinic acid	0.00	Down	Oxidative phosphorylation
adenosine diphosphate (ADP)	0.00	Up	Oxidative phosphorylation
L-Tyrosine	0.00	Up	Phenylalanine metabolism
Trans-Cinnamate	0.00	Up	Phenylalanine metabolism
Phenyllactate	0.00	Up	Phenylalanine metabolism
Prostaglandin F2α	0.01	Up	Arachidonic acid metabolism

**Table 6:** Examining the varied metabolites and associated pathways in the livers between the LPS and LAEE groups.

Metabolite	*p* Value	Variation	Pathway
Succinic acid	0.018	Up	Oxidative phosphorylation
adenosine diphosphate (ADP)	0.04	Down	Oxidative phosphorylation
Phenylpyruvic acid	0.01	Up	Phenylalanine metabolism
Phenyllactate	0.04	Down	Phenylalanine metabolism
L-Arogenate	0.03	Down	Phenylalanine metabolism
Prostaglandin F2α	0.04	Down	Arachidonic acid metabolism

## Data Availability

The unprocessed metabolomics information is stored in the MetaboLights public archive, accessible under the code MTBLS9102. The unprocessed sequence information has been forwarded to the NCBI SRA (PRJNA1051968).
